# New Right Bundle Branch Block: A Benign Variant or an Ominous Sign?

**DOI:** 10.7759/cureus.86103

**Published:** 2025-06-15

**Authors:** Thaw Myint Thu, Ahmed Hegazy, Luke Dancy

**Affiliations:** 1 Cardiology, Medway Maritime Hospital, Gillingham, GBR; 2 Acute Medicine, Medway Maritime Hospital, Gillingham, GBR

**Keywords:** : acute coronary syndrome, coronary artery angiogram, myocardial infarction type 1, non-st elevation myocardial infraction, right bundle branch block

## Abstract

A gentleman in his 80s presented to the hospital with several hours of ongoing, dull-aching chest pain radiating to his left arm. The initial electrocardiogram (ECG) showed atrial fibrillation (AF), bifascicular block (right bundle branch block (RBBB) and left anterior fascicular block), and concordant 0.5 mm ST-segment elevation in leads V1-V3. The repeated ECGs at one-hour and three-hour intervals showed similar findings. The initial high-sensitivity troponin (HS-troponin) level was 2672.7 ng/L. He was triaged as non-ST-segment elevation acute coronary syndrome (NSTE-ACS). However, his ECGs repeated on the next day showed AF, bifascicular block, and ST-segment elevation in leads V2-V4, I, and augmented vector left (aVL) alongside a significant troponin surge to 25,951 ng/L. The emergency coronary angiogram uncovered severe coronary artery disease that warranted percutaneous coronary intervention (PCI).

## Introduction

Nearly 66,000 people in the UK die from acute coronary syndrome (ACS) every year, and the incidence of ACS is noted to be around 2.3 million [1]. In-hospital mortality of ST elevation myocardial ischaemia (STEMI) ranges from 4% to 6% in high-income countries with rapid percutaneous coronary intervention (PCI) access, which is significantly lower than the number in the past due to improvements in early reperfusion coronary intervention [2]. According to the latest European Cardiology Society’s (ESC) ACS guidelines (2023), the recommendation of primary revascularisation intervention mainly targets two cohorts: patients presenting with ST elevation changes on electrocardiograms (ECGs) and unstable patients without ST elevation [3]. However, it is pertinent to be vigilant about acute conduction abnormalities when it comes to triaging and managing ACS patients, since the new changes might suggest significant ongoing ischaemia indicative of emergency revascularisation. In this report, we highlight the importance of conduction abnormality secondary to acute occlusive myocardial infarction. We present a case of ACS with a right bundle branch block (RBBB) and left axis deviation to emphasise the importance of awareness in triaging ACS patients with conduction abnormalities.

## Case presentation

**Figure 1:**
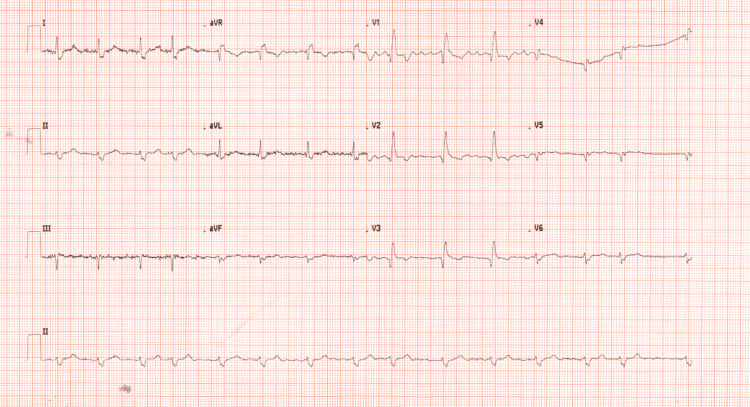
ECG showing atrial fibrillation (AF) and bifascicular block (RBBB + left axis deviation)-suggestive of high-risk ACS with anterior ischaemia ECG: electrocardiogram; RBBB: right bundle branch block;  ACS: acute coronary syndrome

A gentleman in his 80s presented to the hospital with several hours of ongoing, dull aching chest pain radiating to his left arm. His past medical history was significant only for hypertension, for which he takes ramipril. Clinical examinations were unremarkable apart from he was restless in pain. The initial ECG (Figure 1) showed atrial fibrillation (AF), left axis deviation, and RBBB. Repeat ECGs performed at one-hour and three-hour intervals showed similar findings. No previous ECGs were available for comparison. Baseline ECG unavailability limited the assessment of the chronicity of conduction abnormalities. The high-sensitivity troponin (HS-troponin) level at presentation was 2672.7 ng/L (Table 1).

**Table 1:** Laboratory trends showing significant troponin elevation and evolving electrolyte abnormalities

Tests (Units)	1^st^ day	2^nd^ day	Normal range
High sensitivity troponin (ng/L)	2672	25951	0-18
CRP (mg/L)	6		0.8-3.0
Hb (g/L)	154	146	130-170
White cell count (*10^9/L)	11.2	13.1	4-11
Platelets (*10^9/L)	147	150	150-400
PT (sec)	18.2		11-13.5
INR	1.6		0.8-1.2
Cholesterol (mmol/L)	5.3		<5
Triglyceride (mmol/L)	0.62		<1.7
HDL (mmol/L)	1.36		>1
LDL (mmol/L)	3.7		<3.36
HbA1c (mmol/mol)	41		15-42
GFR (mL/min/1.73 m^2^)	>90	84	>90
Urea (mmol/L)	6.3	5.9	2.1-8.5
Creatinine (umol/L)	64	76	64-104
Na (mmol/L)	136	130	136-145
K (mmol/L)	3.7	4.3	3.5 - 5

The case was discussed with two primary PCI centres, which classified it as a non-ST-elevation myocardial ischaemia (NSTEMI) and recommended dual antiplatelet therapy, fondaparinux, and delayed coronary angiography. Thus, the advised management was commenced. 

**Figure 2:**
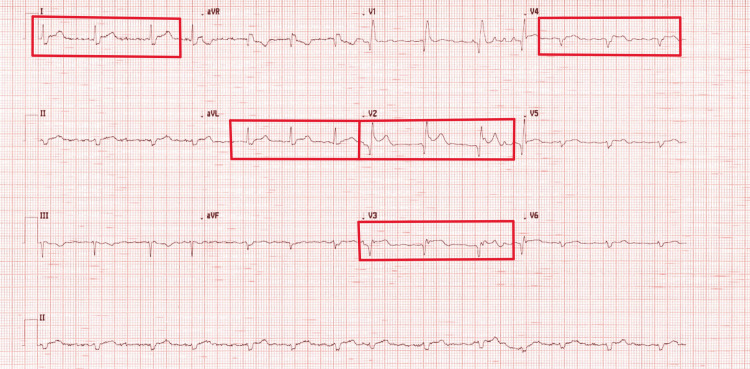
ECG showing atrial fibrillation (AF), bifascicular block (RBBB + left axis deviation), and ST-segment elevation in leads V2-V4, I, and aVL (in red)-showing anterolateral STEMI ECG: electrocardiogram; RBBB: right bundle branch block; aVL: augmented vector left; STEMI: ST-elevation myocardial ischaemia

The transthoracic echocardiogram showed mid- to apical septum, left ventricular (LV) apex, antero-septum, and mid- to apical anterior wall akinesia, and estimated left ventricular ejection fraction (LVEF) of 35-40%. The following day, he still complained of chest pain despite opioid analgesia. An ECG (Figure 2) showed atrial fibrillation (AF), bifascicular block (RBBB + left axis deviation), and ST-segment elevation in leads V2-V4, I, and aVL. He was immediately transferred to the cardiac catheterisation laboratory. The HS-troponin level increased to 25,951 ng/L (Table 1).

**Figure 3:**
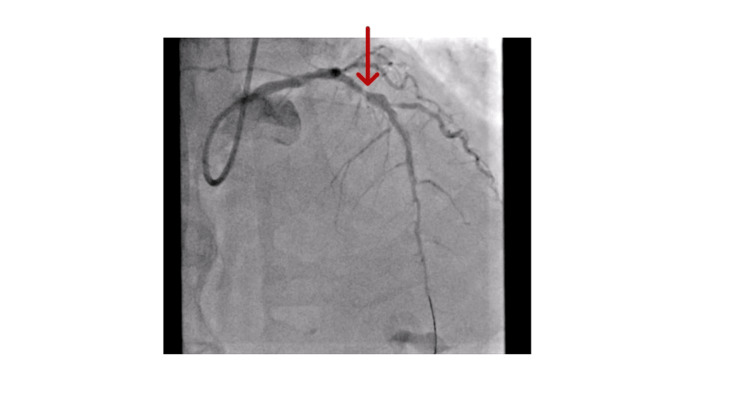
Coronary angiogram picture showing left anterior descending artery (LAD) mid-course occlusion (red arrow)

**Figure 4:**
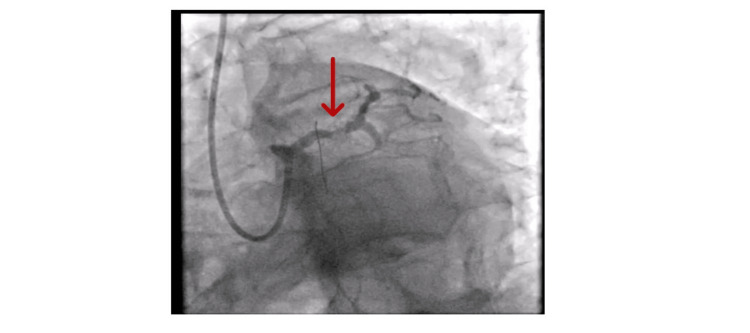
Coronary angiogram picture showing left main stem (LMS) severe mid-distal lesion (red arrow)

Coronary angiography (Figures 3-4) showed left main stem (LMS) severe mid-distal lesion, left anterior descending artery (LAD) mid-course occlusion, left circumflex artery (LCx) proximal chronic total occlusion (CTO), and right coronary artery (RCA) moderate diffuse plaque in a dominant vessel. Given the left main disease, ongoing pain, and anterior ST-segment elevation, a multidisciplinary team (MDT) discussion with two interventional cardiologists resulted in a consensus to perform PCI to the LMS and LAD. 

**Figure 5:**
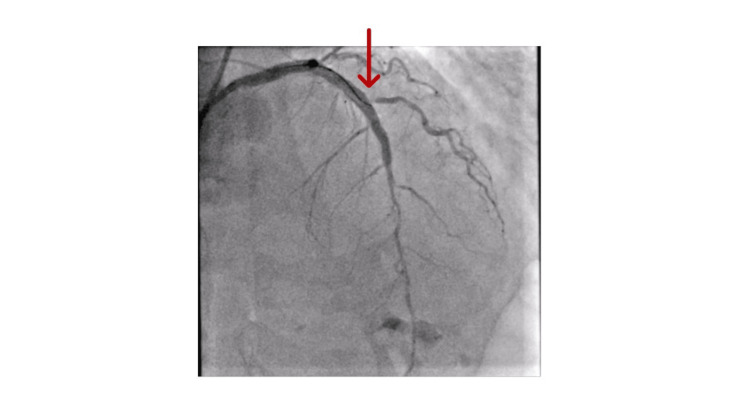
Successful reperfusion to the left anterior descending artery after stenting (red arrow)

The procedure was a successful PCI to the LMS and LAD with implantation of two overlapping Onyx drug-eluting stents (DES) (Figure 5). Left ventricular viability assessment was planned prior to further discussion regarding the need for complete revascularisation. 

Over the following days, he made a good recovery as an inpatient, with resolution of his chest pain. He was discharged with secondary prevention measures for coronary artery disease, heart failure medication, and a direct oral anticoagulant (DOAC) for atrial fibrillation.

## Discussion

The presence of a BBB, not noted on previous ECGs, in the setting of suspected ACS has long been associated with higher mortality [4,5]. The current European Society of Cardiology (ESC) guidelines (2023) define RBBB as QRS duration greater than 120 ms, (rsR’) “bunny ear” pattern in the anterior precordial leads (leads V1-V3), and slurred S waves in leads I, aVL, and frequently V5 and V6 and recommend that patients presenting with a new RBBB and clinical features suggestive of ACS should be considered for urgent reperfusion therapy due to their high-risk status [3]. This stems from accumulating evidence that new RBBB can reflect a proximal coronary occlusion, particularly involving the LAD artery, and may mask or mimic ST-segment elevation, implicating a delay in timely diagnosis [5]. 

In a large retrospective study involving 6,742 patients with acute myocardial infarction (AMI), 6.3% of the studied population were found to have RBBB. Among these, complete occlusion of the infarct-related artery was more common, and these patients were more likely to receive primary PCI compared to those with AMI and left bundle branch block (LBBB). Furthermore, the patients with AMI and RBBB exhibited the highest in-hospital mortality rate among all ECG presentations of AMI, underscoring the gravity of this finding [5]. 

An even more ominous variant, the qRBBB pattern characterised by the combination of pathological Q waves in the anterior leads and RBBB has been suggested to be linked to occlusions of the ostial LAD or left main stem artery [6]. These anatomical lesions carry a high mortality risk due to the extent of myocardium at risk and the potential for hemodynamic compromise. Studies have suggested that qRBBB may serve as an early marker of extensive anterior myocardial infarction and should prompt emergent invasive management. Further studies with larger sample sizes are warranted to more comprehensively investigate this pattern [6, 7]. 

While some research suggests that isolated BBB patterns, in the absence of ST-segment elevation, do not always confer a higher in-hospital mortality compared to STEMI, patients with BBB often present with more severe clinical characteristics such as advanced age, reduced left ventricular function, and greater comorbidity burden [8]. These factors support aggressive early intervention, even in the absence of classic ST elevation. 

Recognizing high-risk ECG patterns such as new-onset RBBB or qRBBB is therefore vital in acute settings such as the emergency department and acute medical admission. Timely identification can facilitate urgent coronary angiography and revascularisation, which have been shown to improve outcomes in this subset of patients [6]. Given the variability in ECG interpretation and its impact on management decisions, increased clinician awareness and continued education are essential. 

In summary, a new RBBB in the context of suspected ACS is not a benign finding. When accompanied by pathological Q waves or suggestive clinical features, it should prompt immediate evaluation for high-risk coronary lesions. Early reperfusion therapy, particularly primary PCI, remains the cornerstone of improving survival and reducing complications in this population [3].

## Conclusions

The current ESC guidelines clearly state that in clinically suspected ACS presentations with ongoing clinical signs suggestive of myocardial ischaemia, the presence of either LBBB or RBBB and/or a paced rhythm impede the accuracy of ST segment assessment on ECG interpretation. Thus, patients presenting with these ECG patterns in combination with signs/symptoms that are highly suspicious for ongoing myocardial ischaemia should be triaged similarly to cohorts with clear ST-segment elevation, regardless of whether the BBB is previously known. In addition, the cohort of patients with ACS who presented with RBBB had higher crude rates of both hospital mortality and major adverse cardiac events (MACE) as compared with patients who presented with ST elevation alone. However, the ESC guidelines also recommend triaging patients with RBBB according to clinical risk of ACS, which highlights the limitation of the data referred to and denounces the evidence gap, especially in groups of patients with other comorbidities, which can mask some of the clinical symptoms of ACS, such as diabetes mellitus. In addition, the current evidence is limited by small sample sizes, retrospective designs, and inconsistent definitions of what constitutes a new conduction abnormality. Few studies distinguish between transient, benign conduction changes and those reflecting malignant ischaemic pathology, and confounding factors such as structural heart disease or electrolyte disturbances are often not accounted for. Clarifying these uncertainties will be essential to improving triage and treatment strategies for ACS patients presenting with atypical ECG findings. All in all, new conduction system abnormalities, particularly the RBBB and bifascicular block, should be recognised as potential indicators of critical coronary occlusions such as left main stem or proximal LAD infarction, especially when accompanied by subtle or equivocal ST changes. Prompt recognition of these patterns is vital in ACS settings, where delays in diagnosis and treatment can significantly impact outcomes.

## Appendices

Abbreviations

ACS: Acute coronary syndrome

AF: Atrial fibrillation

ALT: Alanine transaminase

AMI: Acute myocardial ischaemia

BBB: Bundle Branch Block

CRP: C-reactive protein

CTO: Chronic total occlusion

DOAC: Direct oral anticoagulant

ECG: Electrocardiogram

ESC: European Society of Cardiology

GFR: Glomerular filtration rate

Hb: Haemoglobin

HDL: High-density lipoprotein

HS troponin: High-sensitivity troponin

INR: International normalised ratio

K: Potassium

LAD: Left anterior descending artery

LBBB: Left bundle branch block

LCx: Left circumflex artery

LDL: Low-density lipoprotein

LMS: Left main stem artery

LV: Left ventricle

LVEF: Left ventricular ejection fraction

Na: Sodium

NSTEMI: Non-ST elevation myocardial ischaemia

OMI: Occlusive myocardial ischaemia

PCI: Percutaneous coronary intervention

PT: Prothrombin time

PTT: Partial thromboplastin time

RBBB: Right bundle branch block

RCA: Right coronary artery

STEMI: ST elevation myocardial ischaemia

WBC: White blood cells
